# The emerging vertebrate model species for neurophysiological studies is *Danionella cerebrum*, new species (Teleostei: Cyprinidae)

**DOI:** 10.1038/s41598-021-97600-0

**Published:** 2021-09-23

**Authors:** Ralf Britz, Kevin W. Conway, Lukas Rüber

**Affiliations:** 1 Senckenberg Natural History Collections Dresden, Museum of Zoology, 01109 Dresden, Germany; 2 Research Associate, Department of Life Sciences, Natural History Museum, London, SW75BD UK; 3 Department of Ecology and Conservation, Biology and Biodiversity Research and Teaching Collections, Texas A&M University, College Station, College Station, TX 77543 USA; 4 Research Associate, Ichthyology, Australian Museum Research Institute, 1 William Street, Sydney, NSW 2010 Australia; 5 Naturhistorisches Museum Bern, 3005 Bern, Switzerland; 6 Aquatic Ecology and Evolution, Institute of Ecology and Evolution, University of Bern, 3012 Bern, Switzerland

**Keywords:** Developmental biology, Zoology

## Abstract

The four described species of *Danionella* are tiny, transparent fishes that mature at sizes between 10–15 mm, and represent some of the most extreme cases of vertebrate progenesis known to date. The miniature adult size and larval appearance of *Danionella*, combined with a diverse behavioral repertoire linked to sound production by males, have established *Danionella* as an important model for neurophysiological studies. The external similarity between the different species of *Danionella* has offered an important challenge to taxonomic identification using traditional external characters, leading to confusion over the identity of the model species. Using combined morphological and molecular taxonomic approaches, we show here that the most extensively studied species of *Danionella* is not *D. translucida*, but represents an undescribed species, *D. cerebrum* n. sp. that is externally almost identical to *D. translucida*, but differs trenchantly in several internal characters. Molecular analyses confirm the distinctiveness of *D. cerebrum* and *D. translucida* and suggest that the two species are not even sister taxa. Analysis of the evolution of sexual dimorphisms associated with the Weberian apparatus reveals significant increases in complexity from the simpler condition found in *D. dracula*, to most complex conditions in *D. cerebrum*, *D. mirifica* and *D. translucida*.

## Introduction

Progenesis is an evolutionary process that speeds up gonad development in relation to somatic development, so that the result is a small organism with larval features yet ripe gonads^1,2^. Prominent vertebrate examples of this type of heterochronic change are the species of the cyprinid genus *Danionella*. So far, four species of the genus *Danionella*, maturing at sizes of 10–15 mm in length and including some of the smallest fishes and vertebrates, have been described from Myanmar and northeastern India^3–6^. Though progenesis may act at the level of an individual character or character complex, leading to a mosaic of paedomorphic features in the adult, *Danionella* is unusual in that it shows organism–wide progenesis or developmental truncation, a heterochronic change that leads to tiny adult organisms with an overall larval appearance^7–10^.

It is this larval condition of their adult skeleton, in which the skull roof is missing and the brain is covered only by skin, that has led various researchers to turn to *Danionella* as the adult vertebrate model organism to study neurophysiological questions by deep imaging their brain activity in vivo^11–14^. Species of the genus *Danionella* are further remarkable in possessing a number of unique morphological novelties in males. These include a redirection of their gut and genital ducts so that both open in between the pelvic fins, a large drumming muscle that originates from expanded bony flanges on the *os suspensorium* of their Weberian apparatus and inserts on a hypertrophied fifth rib, and a conical drumming cartilage that probably works like a drumstick on the anterior swimbladder chamber^4–6,9^. This highly complex vocalization apparatus has been identified as a promising organ system to study neurophysiologically^11^.

While the organism-wide progenetic nature of *Danionella* has been the prerequisite for their establishment as neurophysiological model organisms, their larval appearance has a downside for systematists: few of the commonly used external character systems are available for study, as colour pattern is highly reduced to a few melanophores, and scales are absent. The four recognized species have thus been distinguished by fin-ray counts and internal anatomy^4–6^ and are difficult to tell apart when alive.

With the two species of *Danionella* that are used in these studies, *D. dracula* and *D. translucida*, at the start of their career as vertebrate models, we began to take a comparative taxonomic and anatomical look at these fishes. The skeletal anatomy of *D. dracula* was already the subject of a monographic study^9^, but information on *D. translucida* is so far limited to the scant original description^3^. When comparing cleared and double stained specimens of *D. translucida* from the type locality and from additional localities around the southern tip of the Rakhine Yoma in Myanmar, we noticed a striking difference in the chondrocranium between individuals from different localities. This led us to compare in detail the skeletal anatomy of these two forms, and the other species of *Danionella,* and to complement our anatomical study with molecular data. Here we show that the organism that has been introduced as a promising model organism for neurophysiological research is not *D. translucida*, but an undescribed species, which we describe here in detail as *Danionella cerebrum* new species. We discuss anatomical differences between *D. cerebrum* new species and the other four species of *Danionella* and comment on the anatomical changes in the Weberian apparatus and associated structures during the evolution of this genus. We conclude by stressing that the study of *Danionella* presents a promising system to understand major morphological changes as the five species show anatomical differences far greater even than between species of only remotely related genera within the subfamily Danioninae.

## Results

### Taxonomy

#### *Danionella cerebrum* new species

Holotype. BMNH 2021.8.30.1, female, 12.6 mm SL, Myanmar, Yangon Division, Hmawbi, roadside canal draining into Thandabin Chaung, 17° 06.200′ N 96° 02.890′ E, Britz et al., 18 Oct 2008.

Paratypes. MTD 39985, 245 specimens, 7.5–12.0 mm SL, same information as holotype. MTD 39986, 20 c&s, 10.5–13.5 mm SL, same information as holotype. ZRC 62210, 36, 7.2–12.4 mm SL, same information as holotype. NRM 71156, 36, 7.7–10.8 mm SL, same information as holotype. USNM 439009, 36, 7.8–12.0 mm SL, same information as holotype. BMNH 20.21.6.2.1-200, 200, 7.2–11.8 mm SL, same information as holotype. MTD 39987, 1, 10.4 mm SL, c&s, Myanmar, Bago Division, Daik U, Daikme Chaung, 17° 48.267′ N 96° 39.826′ E, Britz et al., 19 Oct 2008. MTD 39988, 10–12 mm SL, 4, unnamed stream 14.4 km NE of Daik U, 17.89314° N 96.56361°E, Britz et al., 19 Oct 2008. MTD 39989, 1 c&s, 11.4 mm SL, unnamed stream 14.4 km NE of Daik U, 17.89314° N 96.56361°E, Britz et al., 19 Oct 2008.

Additional material (non type): MTD 39990, 29 specimens, 7.8–11.5 mm SL, aquarium shop in Bago, reportedly from Thandabin Chaung, Britz et al., 19 Oct 2008. MTD 39991, 8 c&s, 8.5–11.3 mm SL, aquarium shop in Bago, reportedly from Thandabin Chaung, Britz et al., 19 Oct 2008.

##### Diagnosis

*Danionella cerebrum* is distinguished from *D. translucida*, *D. dracula*, and *D. priapus* by the number of anal-fin rays (15–18 vs. 12–15 in *D. translucida*, 12–14 in *D. dracula*, 20–21 in *D. priapus*, Table 1). It is further distinguished from *D. mirifica*, *D. dracula*, and *D. priapus* by fewer vertebrae (33–35 vs. 36–38, Table 1), from *D. priapus* and *D. dracula* by fewer pectoral-fin rays (6 vs. 8 in *D. priapus* and 7 in *D. dracula*, Table 1), from *D. translucida* and *D. dracula* by the presence of a ventromedially directed cartilage flange from the *taenia marginalis anterior* that approaches the *trabecula communis* (vs. absence, Fig. 2c,d), and from *D. dracula* by the presence in the male of bony flanges on the outer arm of the *os suspensorium* and a connection of these to the lateral process of vertebra 2 (vs. absence of flanges and of connection to second lateral process), the presence of a maxillo-mandibular cartilage (vs. absence), the absence of odontoid processes in the male (vs. presence), more anal-fin pterygiophores (14–17 vs. 11–13, Table 1), more principal caudal fin rays (9 + 9 vs. 8 + 8, Table 1) and fewer pelvic-fin rays (5 vs. 6, Table 1). *Danionella cerebrum* can be further distinguished from the similar syntopically living *D. translucida*, by the last dorsal-fin ray inserted opposite to the last anal-fin ray (vs. last dorsal-fin ray inserted posterior to last anal-fin ray, Fig. 2a,b), by the last anal-fin pterygiophore inserted in front of haemal spine of vertebra 22–24 (vs. 19–21), by the lateral process of the second vertebra blade-like (vs. axe shaped, Fig. 2g,h), and by the distal tip of the fused inner arms of the *ossa suspensoria* bifurcated (vs. single, Fig. 2e,f) and not reaching the middle of the anterior swimbladder (vs. curving around and reaching middle of anterior swimbladder, Fig. 2e,f).

**Table 1:** Comparative meristic information of the five species of *Danionella*. Information for *D. mirifica*, *D. dracula* and *D. priapus* taken from Britz^4,5^ and Britz et al.^6,8^. Counts of *D. cerebrum* (n = 32) based on MTD 39986, MTD 39987 and MTD 39989, those of *D. translucida* (n = 28) on MTD 39992, MTD 39993.

	*D. cerebrum*	*D. translucida*	*D. mirifica*	*D. dracula*	*D. priapus*
Dorsal-fin rays	8–9	8–9	8–10	7–8	9–10
Anal-fin rays	15–18	12–15	17–20	12–14	20–21
Principal caudal-fin rays	9 + 9	8–9 + 8–9	9 + 9	7–8 + 7–8	9 + 9
Dorsal procurrent caudal-fin rays	5–8	5–7	5–6	3–4	7–8
Ventral procurrent caudal-fin rays	5–8	3–7	6–7	2–3	6–8
Pectoral-fin rays	6	6–7	6–7	7	8
Pelvic-fin rays	5	5	5	6	5
Vertebrae	33–35	32–34	36–37	36–37	37–38
Abdominal	13–14	12–14	14–15	15–16	15–16
Caudal	19–21	19–21	21–22	20–22	22–23
Ribs	7–8	7–8	7–9	8	10
Anal-fin pterygiophores	14–17	11–14	16–19	11–13	19–20
Insertion of anal-fin pterygiophores	22–24	19–21	23–25	23–24	27–28

##### Description

**Figure 1:**
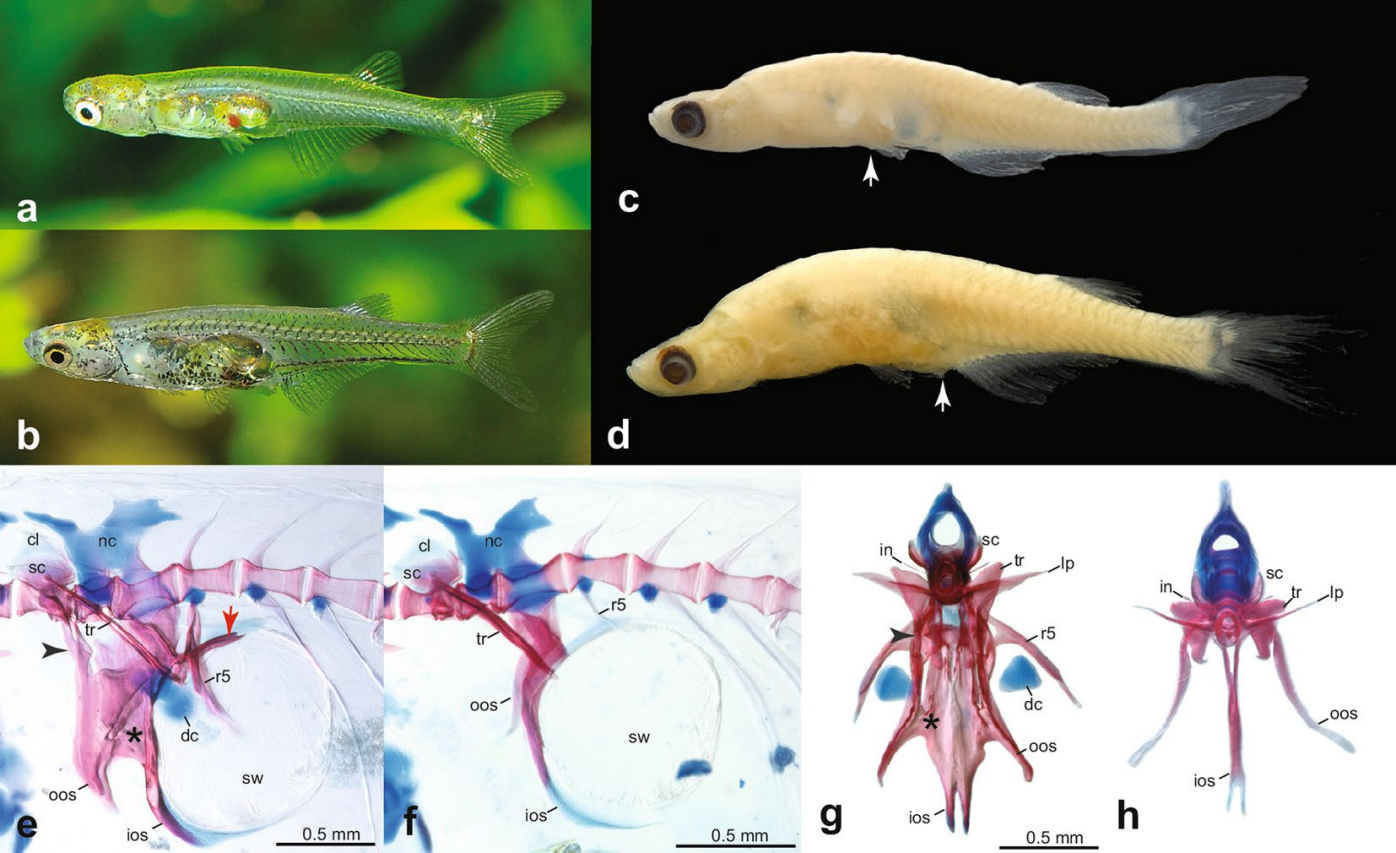
*Danionella cerebrum*. (**a**) male (ca. 10 mm SL) and (**b**) female (ca. 12 mm SL) in life, not preserved; note yellowish chromatophores dorsally on head, melanophores scattered in rows on body in both sexes, and eggs covered by large melanophores in female; (**c**) MTD 39985, paratype, 10.4 mm SL, male and (**d**) BMNH 2021.8.30.1, holotype, 12.6 mm SL, female (below), white arrows mark position of vent, which is shifted anteriorly to the pelvic fins in males; (**e**) Weberian apparatus in male, MTD 39992, paratype, 11.7 mm SL and (**f**) female, MTD 39992, paratype, 11.8 mm SL, in lateral view; the same in male (**g**) and female (**h**) in frontal view; (**e**) and (**g**) black arrowhead marks connection between lateral process and outer arm of *os suspensorium*, star marks connecting flanges between inner and outer arms of *os suspensorium* and red arrow marks posterior extension of inner arm of *os suspensorium* covering swimbladder dorsally. Abbreviations: cl, claustrum; dc, drumming cartilage; ios, inner arm of *os suspensorium*; nc, neural complex; oos, outer arm of *os suspensorium*; r, rib; sc, scaphium; sw, swimbladder.

Maximum known size 13.5 mm SL. General body shape illustrated in Fig. 1a–d. Morphometric information based on 10 specimens is provided in Table 2. Head and eye are large; mouth supraterminal. Nostrils well developed. Lateral line canals and pores on head and body absent. Body elongate with a short dorsal fin, situated opposite to posterior half of long anal fin. Tip of dorsal fin situated posterior to a vertical line through tip of anal fin. Caudal fin furcate with remnants of larval-fin fold in front of its dorsal and ventral margins. A remnant of pre-anal larval-fin fold present in adult females, absent in adult males. Anus and genital papilla of mature males located between pelvic fins, at normal position in front of anal fin in females and in immature males between pelvic and anal fins. A window (pseudotympanum) present in body musculature at lateral side of anterior swim bladder chamber, rendering its pigmented surface visible. Scales absent.

**Table 2:** Morphometric information of 10 specimens (5 males, 5 females) of *Danionella cerebrum* (BMNH 2021.8.30.1, MTD 39985), information of holotype in parentheses behind range.

	Range	Average	Standard deviation
Standard length (SL) in mm	10.0–12.6 (12.6)		
**In % SL**			
Head length (HL)	20–21.9 (21.4)	21.0	0.6
Predorsal length	66.1–71.4 (69.6)	69.6	1.4
Preanal length	52.5–56.3 (54.5)	54.5	1.3
Body depth at vent	15.4–19.0 (19.0)	17.3	1.1
Caudal peduncle depth	7.7–9.0 (8.7)	8.4	0.4
**In % HL**			
Eye diameter	28.0–31.8 (29.6)	30.0	1.8
Snout length	20.0–24.0 (22.2)	22.6	1.4

Vertebrae totaling 33(7), 34(23), 35(2), abdominal vertebrae 13(15), 14(16) or 15(1); caudal vertebrae 19(4), 20(17) or 21(11). Ribs present on vertebrae 5–11(30) or 5–12(2). Rib on vertebra 5 dimorphic, stout and well ossified in male, feeble and poorly ossified in female. Dorsal-fin rays 8(31) or 9(1), first two fin rays unbranched (32) and last unbranched (25) or branched (7). Dorsal-fin pterygiophores 7(31) or 8(1). First dorsal-fin pterygiophore inserted behind neural spine of vertebra 18(9), 19(19), 20(3) or 21(1), and last in front of neural spine of vertebra 22(1), 23(22), 24(8) or 25(1). Anal-fin rays 15(8), 16(15), 17(7) or 18(1) with first two rays unbranched (32) and last unbranched (26) or branched (6). Number of anal-fin pterygiophores in front of first haemal spine: 0(7), 1(11) or 2(15). Last anal-fin pterygiophore inserted in front of haemal spine of vertebra 22(14), 23(15) or 24(3). Principal caudal-fin rays 9 + 9(32) plus 5(2), 6(8), 7(16) or 8 (6) dorsal and 5(1), 6(18), 7(12) or 8(1) ventral procurrent rays. Pectoral-fin rays 6(32) and pelvic-fin rays 5(32).

No visible pigmentation in preserved specimens, except a line above anal-fin base in some specimens. In life, body colourless and largely translucent (Fig. 1a,b), except for a number of melanophores and yellowish colouration covering dorsal surface of skull. Melanophore pattern including an irregular scattering on top and sides of head, a row following the posterior margin of shoulder girdle, oblique melanophore rows along neural and haemal arches and spines in deeper layers of body, a horizontal row along insertion of anal-fin muscles starting above vent and extending posteriorly along caudal peduncle to anterior ventral procurrent rays, a row of melanophores at base of anal fin itself and along first anal-fin rays, and melanophores marking end of the hypural plate. Females with eggs, with numerous, large melanophores in lining of abdominal wall.

**Figure 2:**
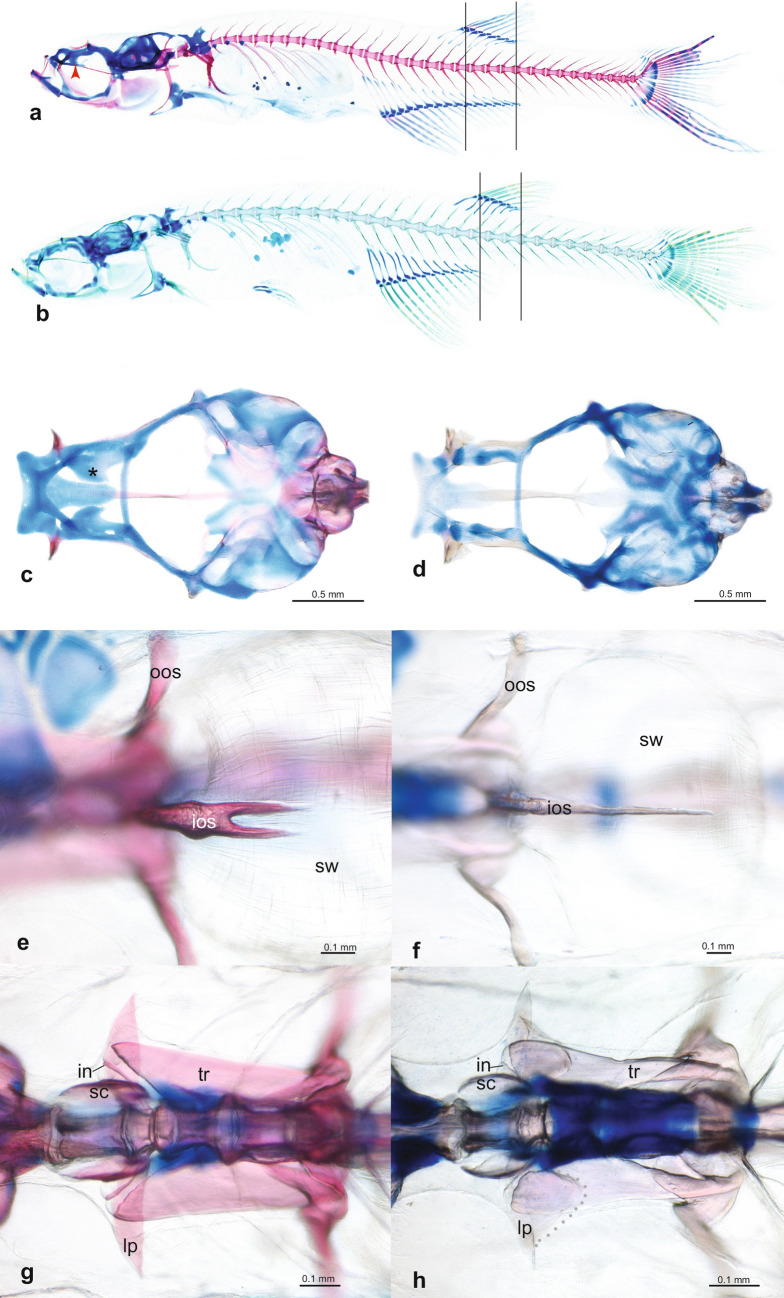
Comparison of skeletal characters of cleared and stained females of *Danionella cerebrum*, and *D. translucida*. Whole skeleton of *D. cerebrum* (**a**), MTD 39986, paratype, 11.5 mm SL and *D. translucida* (**b**), MTD 39992, 13.1 mm SL in lateral view, illustrating differences in relative position of dorsal and anal fins; vertical lines mark base of anteriormost and posteriormost dorsal-fin ray, respectively, in relation to anal fin. Neurocranium of *D. cerebrum*, MTD 39986, paratype,13.5 mm (**c**) and *D. translucida* (**d**), MTD 39992, 11.2 mm SL, in dorsal view, star marks ventromedial cartilage flange in (**c**), which is absent in (**d**). Tip of fused inner arms of *ossa suspensoria* in *D. cerebrum* (**e**) and *D. translucida* (**f**), in ventral view; note bifurcated tip in (**e**) and single tip in (**f**). Vertebrae of Weberian apparatus in *D. cerebrum* (**g**) and *D. translucida* (**h**), in dorsal view; note caudally expanded lateral process in **(h)**, margin marked by line of grey dots. Abbreviations: in, intercalarium; ios, inner arm of *os suspensorium*; lp, lateral process of second vertebra; oos, outer arm of *os suspensorium*; r, rib; sc, scaphium; sw, swimbladder; tr, tripus.

The cleared and double stained specimens (Fig. 2a,b) revealed that, as in other species of the genus *Danionella*, the skull, hyopalatine arch, gill arches, endoskeletal shoulder girdle and pterygiophores are mostly cartilaginous with only thin perichondral ossifications giving the skeleton an overall larval appearance. The following bones are absent in *D. cerebrum*: kinethmoid, preethmoid, vomer, nasal, parietal, intercalar, extrascapular, infraorbitals 2–5, angular, ectopterygoid, metapterygoid, urohyal, hypobranchials 1–3, posttemporal, postcleithrum, mesocoracoid, pectoral radials 3–4, pelvic radials 1–3, all supraneurals behind supraneural 3, epineurals, epipleurals, uroneural 2, and scales. Exceptions to this theme of bone loss and reduction in the skull are the heavily ossified and toothed ceratobranchial 5, which is essential in food processing in conjunction with the well-ossified basioccipital, which carries the masticatory plate that ceratobranchial 5 works against. The basioccipital along with the equally well-ossified exoccipital houses the intracranial part of the Weberian apparatus (*sinus impar*, capsules for *lagena* and *asteriscus*). Especially well-developed are also the Weberian ossicles and the *os suspensorium*, whose inner arms are fused in the midline with a bifurcated tip. There is a strong sexual dimorphism in the *os suspensorium* with males having the outer and inner arms more massively developed, the inner arms covering the roof of the swimbladder via posterior processes, the inner and outer arms connected via a broad bony flange and having the outer arms connected to the transverse processes of the second vertebra by a bony process. In addition males have a drumming cartilage associated with the fifth rib and swimbladder. Females lack all these modifications. Also the fifth rib is sexually dimorphic, stout and well ossified in the male and with a ventromedially directed process near its base, and feeble and poorly ossified in the female and lacking such a process.

##### Etymology

The species name *cerebrum*, Latin for brain, a noun in apposition, makes reference to the fact that this fish with one of the smallest adult brains among vertebrates has become a promising new model species for neurophysiological studies.

##### Distribution

**Figure 3:**
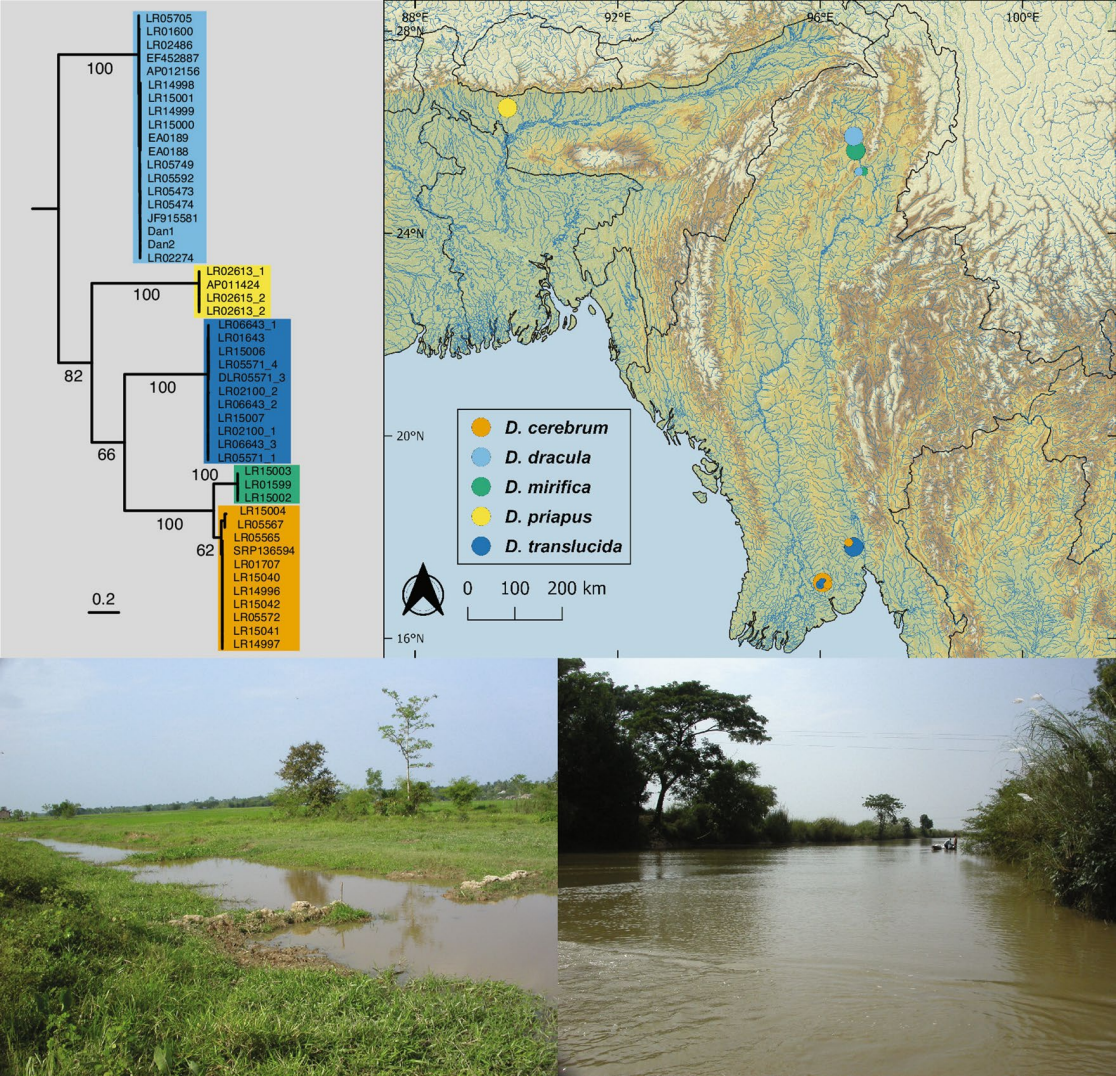
Raxml tree for the coxI gene of the five species of *Danionella* (upper left) from different sampling locations (data set 1) and map (upper right) showing type localities (large circles) and locations of additional samples (small circles). Note that both species, *D. cerebrum* and *D. translucida*, co-occur at each other’s type locality. Roadside canal at Hmawbi (lower left), type locality of *D. cerebrum*, and Daikme Chaung (lower right), type locality of *D. translucida*, illustrating the typical turbid streams in which these two species occur. Map created with QGIS version 3.8.3-Zanzibar (http://www.qgis.org).

*Danionella cerebrum* is known from a number of streams on the southern and eastern slopes of the Bago Yoma mountain range (Fig. 3) of Myanmar: Thandabin Chaung and Bala Chaung in Yangon Division, and from Daikme Chaung (type locality of *Danionella translucida*) and an unnamed stream northwest of Daikme Chaung in Bago Division.

##### Habitat

All water bodies in which *Danionella cerebrum* was found, are turbid low altitude streams (Fig. 3) with visible flow, surface temperatures of around 30 °C, pH 7.4–7.5 and soft water of 20–100 micro Siemens. *Danionella cerebrum* was not found at the surface, but at a depth below ca. 30 cm where the water is significantly cooler (ca. 25 °C). The species was abundant at the type locality in Hmawbi on 18 Oct 2008, where it co-occurred with *D. translucida* (as evidenced by c&s specimen and DNA sample), which appears to have been less common (a single c&s *D. translucida* among 28 *D. cerebrum*). At Daikme Chaung (Fig. 3), the type locality of *D. translucida*, *D. cerebrum* was uncommon (1 out of 27 c&s specimens of *Danionella*) on 19 Aug 2008, but *D. translucida* was abundant.

##### Molecular results

**Figure 4:**
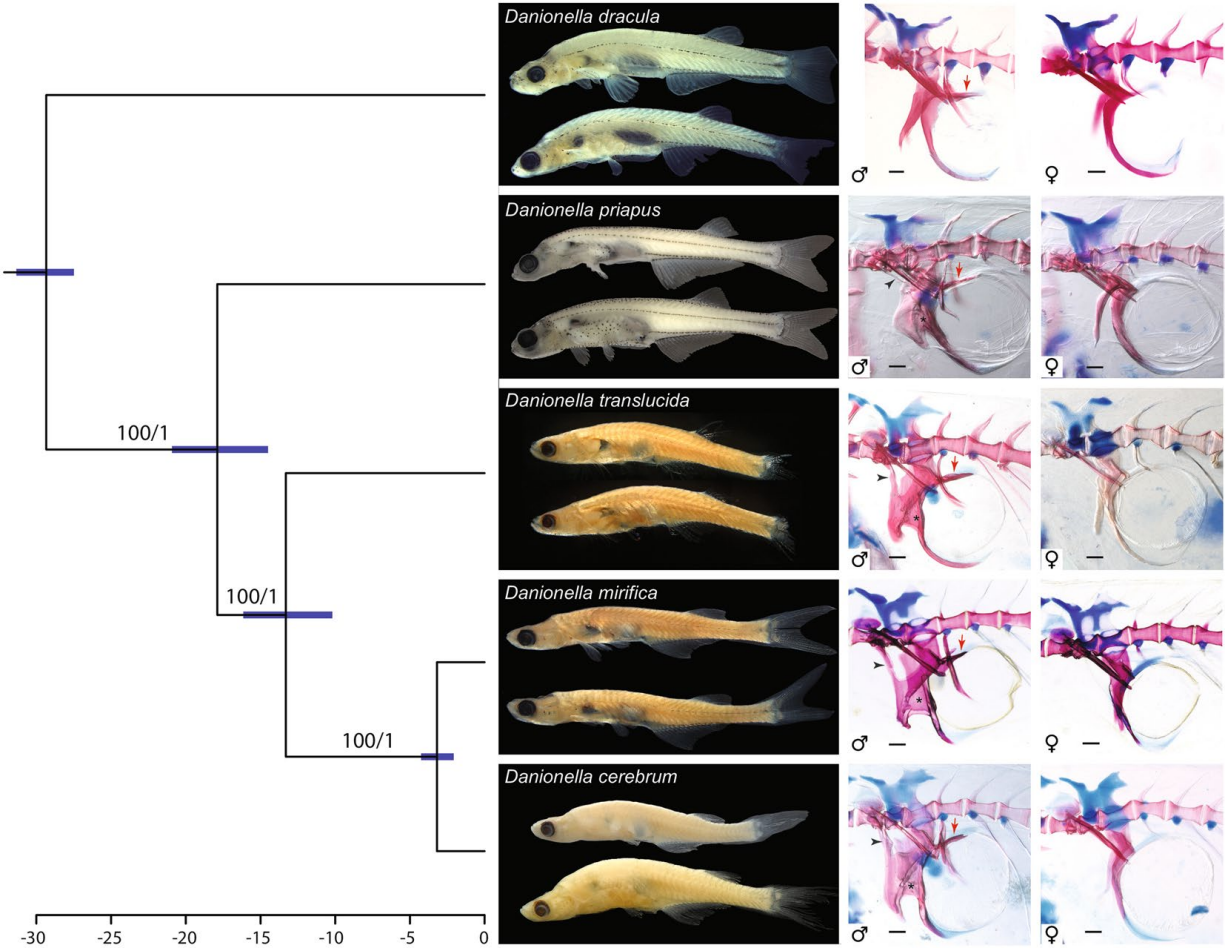
Timetree of the five species of *Danionella* illustrating relationships of *D. cerebrum* (left), differences in external appearance of preserved specimens (middle), and sexual dimorphisms in the skeleton of the Weberian apparatus (right, double column) in cleared and double stained specimens. Preserved specimens (middle) from top: *Danionella dracula*, BMNH 2008.1.1.1, male, holotype, BMNH.1.1.2–99, female, paratype, *D. priapus*, BMNH 2009.9.9.1, male, holotype, BMNH 2009.9.9.2–37, paratype, female; *D. translucida* NRM 32235, male and female paratypes; *D. mirifica*, USNM 372848, male and female paratypes; *D. cerebrum*, MTD 39985, male, paratype, BMNH 2021.8.30.1, female, holotype. Cleared and stained specimens (scale bar 0.1 mm), males, left column from top: *D. dracula*, BMNH 2008.1.1.100–119, 16.2 mm; *D. priapus*, BMNH 2009.9.9.38–43, 16.5 mm; *D. translucida*, MTD 39992, 9.8 mm; *D. mirifica*, USNM 372848, 13.2 mm; *D. cerebrum*, MTD 39986, 11.7 mm; black arrowheads mark connection between lateral process and outer arm of *os suspensorium*, black stars mark connecting flanges between inner and outer arms of *os suspensorium,* and red arrows marks posterior extension of inner arm of *os suspensorium* covering swimbladder dorsally. Females, right column from top: *D. dracula*, BMNH 2008.1.1.100–119, 14.7 mm; *D. priapus*, BMNH 2009.9.9.38–43, 14.8 mm; *D. translucida*, MTD 39992, 11.2 mm; *D. mirifica*, USNM 372848, 13.2 mm, *D. cerebrum*, MTD 39986, 11.7 mm.

The 12 individuals of *Danionella cerebrum* and *D. mirifica* sequenced for the cytochrome coxidase subunit I (coxI) gene had unique adenin insertions at positions 95 and 624 of the coxI alignment leading to frame shifts and premature stop codons as previously reported for the gobioid *Parapocryptes serperaster*^15^, the significance of which is unknown. Interestingly, we were also able to identify two adenin insertions in the coxI gene in the whole genome sequence of *D. cerebrum* provided by Kadobianskyi et al.^16^ (GenBank accession SRMA00000000 as *D. translucida*) as well as the expected mt DNA tRNAs flanking the coxI, hundreds of base pairs up and downstream of the coxI region sequenced in our study, suggesting a genuine mitochondrial origin of our coxI sequences rather than a nuclear pseudogene (NUMT). For the phylogenetic analyses, however, the two insertions were excluded. In order to make sure that our phylogenetic analyses were not affected by the unlikely inclusion of NUMTs, we also conducted phylogenetic analyses based on an additional mitochondrial marker (16S rRNA) and three nuclear DNA markers (erg2b, rag1, rho). The different loci resulted in the same tree topology for the five species of *Danionella* but showed different support values (Figs. 3, 4, Electronic Supplementary Fig. 2).

Our initial result based on morphological information that the model organism used in Schulze et al.^11^ is not *Danionella translucida*, but a separate species, *D. cerebrum*, is supported by our molecular analysis. Samples from three different localities around the southern end of the Bago Yoma mountain range, including the type locality of *D. cerebrum*, clustered with samples obtained from the stock kept at Bolton Aquarium, from which the individuals used in Schulze et al.^11^, in Penalva et al.^12^ and Kadobiansky et al.^16^ originated and with the sample used in Britz et al.^6^ and labeled *Danionella* sp. “South Myanmar” LR1707.

We found that *Danionella cerebrum* differs significantly from its close congeners and the uncorrected p-distances in the coxI gene between this species and *D. translucida* (Electronic Supplementary Table 2), the species with which it has been previously confused, ranged from 22.2–22.9%. Even between *D. cerebrum* and its closest relative *D. mirifica*, p-distances still range from 10.2–10.9%.

We recovered the following topology among the five species of *Danionella* independent of the genes analysed (Fig. 4): (*D. dracula*,(*D. priapus*,(*D. translucida*,(*D. mirifica*,*D. cerebrum*)))). The age for the split of *D. priapus* from the remaining three taxa was estimated at ~ 17.9 MYA (95% HPD 14.5–20.9) and the split of *D. translucida* from *D. mirifica* + *D. cerebrum* at ~ 13.3 MYA (95% HPD 10.1–16.1). The sister taxa *D. mirifica* and *D. cerebrum* split at ~ 3.2 MYA (95% HPD 2.0–4.2).

## Discussion

Alive or preserved, *Danionella cerebrum* is difficult to distinguish from *D. translucida*, because the two look very similar and the distinguishing count of anal-fin rays is challenging to obtain, as the most posterior fin rays are very small and often pressed close to the body rendering their number extremely difficult to count. Reliable anal-fin ray counts, however, can be easily obtained in c&s specimens (see Fig. 2a,b) and distinguish the majority of specimens of both species, with 15–18 rays in *D. cerebrum*, but 12–14, rarely 15, rays in *D. translucida*. The difficulty to distinguish the two species with external characters is contrasted by marked differences in their internal anatomy. The most obvious one that led us to reinvestigate our samples of *Danionella* from the Bago Yoma is the flange of cartilage that extends ventromedially from the *taenia marginalis anterior* towards the *trabecula communis* (Fig. 2c,d). Its posterior and posteroventral margin is covered by the perichondrally ossified orbitosphenoid, a bone that is restricted to the *taenia marginalis anterior* in *D. translucida* (Fig. 2d). There is also a difference in the medially fused *ossa suspensoria*, the tip of which is bifurcated and ends at the anterior curvature of the anterior swim bladder chamber in *D. cerebrum*, but is pointed in *D. translucida* and ends in a single tip at the middle of the ventral curvature of the swimbladder chamber (Fig. 2e,f)*.* A third clear difference is in the shape of the lateral process of the second centrum (Fig. 2g,h). This is blade-like in *D. cerebrum* (Fig. 2g) but has a bulging axe-shape in *D. translucida* (Fig. 2h). A less conspicuous though consistent difference is the presence in *D. cerebrum* of a small medioventrally directed process at the base of the fifth rib in the male (Fig. 1g), which potentially plays a role in the production of sound (see below). Such a process is absent in *D. translucida*.

Like the other species of *Danionella*, *D. cerebrum* shows a conspicuous sexual dimorphism putatively related to the production of sound. In mature males, the *os suspensorium* is greatly expanded by a lateral bone flange that bridges the gap between the outer and inner arms and an anterior flange that originates from the anterior margin of the outer arm. This anterior flange is confluent with an anterior process, which in turn is fused to the lateral process of the second centrum. This forms a large rigid basket-like structure in front of the anterior swimbladder chamber. A large drumming muscle originates from these flanges and inserts on the enlarged fifth rib and covers the large conical drumming cartilage like a cup. This apparatus has been hypothesized to be related in the production of sounds that aid in intraspecific communication of 60 and 120 Hz and amplitudes of around 140db with a duration of tens of milliseconds to minutes^11^. Females do not possess these additional flanges, the drumming muscle or the drumming cartilage and their fifth rib is smaller and only poorly ossified.

The most striking sexual dimorphism in *Danionella* is developed in *D. dracula*, in which mature males have huge odontoid fangs that resemble teeth in their arrangement and appearance^6^, while in other species of *Danionella* including *D. cerebrum*, the head skeleton and jaws are not sexually dimorphic.

A sexual dimorphism that is developed in all species of *Danionella* concerns the skeleton of the Weberian apparatus. Roberts^3^ illustrated the skeleton of the Weberian apparatus in a male of *D. translucida*, but he seems to have been unaware of its dimorphic structure in males and females. This was later clarified by Britz^4^ in *Danionella mirifica*, and the sexually dimorphic anatomy of the Weberian apparatus was subsequently also described for *D. dracula*^6,9^ and *D. priapus*^5^.

A comparison of the *os suspensorium* and the associated skeletal structures responsible for sound production shows that there is an increase in complexity of the apparatus and its sexually dimorphic structure during the evolution of the genus *Danionella* (Fig. 4). All species of *Danionella* share the presence of a drumming muscle and drumming cartilage associated with a sexually dimorphic stouter fifth rib and *os suspensorium*, all putative synapomorphies of the genus. However, in *Danionella dracula*, the sister taxon to all other *Danionella*^6^ (Fig. 4), the apparatus of males is similar to that of females, as the anterior flange on the outer arm, its connection to the lateral process of the second vertebra, and the flange connecting outer and inner arms are all missing (Fig. 4). Its main dimorphism is thus the robustness of the different bones, which are wider, better ossified and stouter in males^9^. This is true of the fifth rib as well as the *os suspensorium*, from which the drumming muscle originates^9^.

Males of the Indian *Danionella priapus* share with *D. translucida*, *D. mirifica*, and *D. cerebrum* the flange connecting both arms of the *os suspensorium*, the anterior bone flange on the outer arm and the connecting process to the lateral process of the second vertebra (Fig. 4), putative synpomorphies of this Indo-Burmese clade. Interestingly some mature males of *D. priapus* lack the process connecting it to the lateral process of the second vertebra and, if present, it is much thinner than in the three Myanmar species of this clade. In contrast all mature males of *D. translucida*, *D. mirifica*, and *D. cerebrum* show a well-developed connecting process between the *os suspensorium* and the lateral process, and their bony flanges on the outer and inner arm are more extensive than in *D. priapus*, potentially providing a larger surface for the origin of the drumming muscle.

The striking structural differences in the arrangement of the Weberian apparatus in males of the different species of *Danionella* may equate to differences in the sounds produced by males. Unfortunately, sound in *Danionella* has been recorded and characterized to date only for a single species, *D. cerebrum* (as *D. translucida*^11^). We expect additional comparative studies on sound production in the genus *Danionella* to demonstrate that species-specific differences in the frequency, amplitude and duration of sounds exist as a result of the differences in anatomical details of the putative sound producing apparatus that we observed.

The five species of *Danionella* are textbook examples for the link between miniaturization via developmental truncation and evolutionary morphological novelty, as proposed by Hanken and Wake^2^ and discussed in detail for this genus by Rüber et al.^17^ and Britz and Conway^9^. Specifically for *Danionella*, Conway et al.^10^ have shown that it is the dramatic disparity in heterochronic shifts between different parts of the skeleton that has resulted in a vertebrate with an open, larval skull roof, but a fully developed Weberian apparatus. Both features render species of *Danionella* ideal candidates as neurophysiological model organisms. Unlike the equally tiny cyprinid species of the genus *Boraras* with more or less the same skeleton as their larger relatives of the Rasborini^18,19^, *Danionella* have a developmentally truncated neurocranium, but a precociously developing Weberian apparatus, which in the latter not only aids in the perception of sound, but also evolved into a unique apparatus for sound production. The remarkable combination of features of *Danionella* sets them apart from other cyprinids of the same or larger size and provide character combinations that are of interest to various vertebrate researchers. Whether miniaturization of the body of *Danionella* and its truncated anatomical condition go hand in hand with a miniaturization of the genome and loss of hox and other developmental genes, as recently demonstrated for the equally miniaturized and developmentally truncated danionine *Paedocypris*^20^ will need to be demonstrated. We expect that in the future *Danionella cerebrum* will become another important vertebrate model organism beyond its current significance for neurophysiological studies.

## Methods

### Morphology

Methods, counts and measurements follow Britz et al.^9^, including the clearing and double staining (c&s). All sizes are provided as standard length (SL). Studied specimens are deposited in the collections of the Senckenberg Naturhistorische Sammlungen Dresden (MTD), the Natural History Museum, London (NHM), the Swedish Museum of Natural History (NRM), Stockholm, and the National Museum of Natural History (USNM) Smithsonian Institution, Washington DC. Because two species of *Danionella* co-occur at the type locality of *D. translucida* and the ranges in anal-fin counts reported by Roberts^3^ indicate that the type series of *D. translucida* may have included two species, it was necessary to verify the taxonomic identity of the holotype of *D. translucida* (NRM 32232). Unfortunately Roberts^3^ only provided a drawing of the holotype with little useful information and we had to request and study a photo of the holotype reproduced herein (Electronic Supplementary Fig. 1). Its close examination confirmed that it has an anal-fin ray count of 13 rays thus matching the diagnostic character state listed herein for our *D. translucida* material. Additional material studied: *Danionella translucida*: MTD 39992, 27 specimens, 8.5–12.2 mm SL, c&s, Myanmar, Bago Division, Daik U, Daikme Chaung, 17° 48.267′ N 96° 39.826′ E, Britz et al., 19 Oct 08. MTD 39993, 1 c&s, 9.7 mm SL, aquarium shop in Bago, reportedly from Thandabin Chaung, Britz et al., 19 Oct 2008. *Danionella mirifica*, USNM 372849, paratypes, 20 c&s, 10–14 mm SL, Myanmar: Kachin State: hill stream, 8 miles from Kamaing on road to Tanai, Aung Myint, 20 Feb 2003. *Danionella priapus*, BMNH 2009.9.9.38–43, 6 c&s, 13.6–15.9 mm SL, India, West Bengal, Jalpaiguri District, Brahmaputra drainage, Jorai River, a tributary of the Sankosh at Laskarpara, outskirts of Barobisha town, 26°28′ 52.3″N, 89° 49′ 29.8″E, M. Das, 2 Apr 2008.

### Molecular

To provide a phylogenetic framework for *Danionella* we assembled different data sets with distinct taxon and/or gene sampling consisting of both newly determined sequences and sequences retrieved from Genbank (Electronic Supplementary Table 1): Data set 1, 47 *Danionella* specimens, coxI DNA barcodes (654 bp); data set 2, 22 *Danionella* specimens, 16 s rRNA (469 bp); data set 3, 10 *Danionella* specimens, erg2b (834 bp); data set 4, 15 *Danionella* specimens, rag1 (1422 bp); data set 5, 14 *Danionella* specimens, rho (894 bp). For all data sets *Danio rerio* was included as outgroup. DNA extraction, PCR amplification, sequencing, sequence assembly and alignment were conducted as detailed in Conte-Grand et al.^21^. PCR amplifications and Sanger sequencing were conducted with the following primers: coxI, FishF2cox1 and FishR1cox1^22^; 16S rRNA, 16S-AL-L and 16S-BR-H^23^; erg2b, E2B-278F and E2B-1117R^24^; rag1, RAG1R1 and RAG1F1^25^; rhodopsin, RH28F^24^ and Rod5R^26^.

Maximum likelihood analyses for data sets 1–5 were conducted with RAxML v7.3.4^27^ under the − f a setting and 1000 bootstrap replicates. The optimal partition schemes for the four data sets consisting of protein coding genes were generated using PartitionFinder 1.0.1^28^ using initial partitions according to codon position. The setting model_selection = BIC and search = greedy was used for the different PartitionFinder runs (models = raxml).

Divergence times for data set 1 were estimated in BEAST v2.6.4^29^. After initial test runs and to ensure convergence and obtain ESS values > 200 we used a strict molecular clock and substitution. Clock and tree models were linked. The Yule process was used as speciation model. Based on the results of Britz et al.^6^, who obtained an age of the most recent common ancestor (MRCA) of *Danionella* of 29.5 (95% confidence interval 27.4–31.9) we used a calibration priors for the MRCA of *Danionella* assuming a normal distribution with a mean of 29.5 and a Sigma of 1.0. Two separate MCMC chain were run for 200 Million iterations sampling every 20’000 steps and were combined in LogCombiner^29^ using a conservative burnin of 50%. Trees were annotated in TreeAnnotator^29^ and visualized in FigTree v1.4.4 (http://tree.bio.ed.ac.uk/software/figtree/).

## Supplementary Information

Supplementary Information.

## Nomenclatural acts

This published work and the nomenclatural acts it contains have been registered in ZooBank, the proposed online registration system for the International Code of Zoological Nomenclature (ICZN). The ZooBankLSIDs (Life Science Identifiers) can be resolved and the associated information viewed through any standard web browser by appending the LSID to the prefix ‘http://zoobank.org/’. The LSIDs for this publication are urn:lsid:zoobank.org:pub: 65198876-420A-4C63-B953-F84C55F3F1FB (article), and urn:lsid:zoobank.org:act: EB6CE17B-2A2E-4773-82A4-8EB7A2D40C45 (*Danionella cerebrum*).
