# Supplementary material for: The emerging vertebrate model species for neurophysiological studies is Danionella cerebrum, new species (Teleostei: Cyprinidae)
Source: Sci Rep. 2021 Sep 23;11:18942. doi: 10.1038/s41598-021-97600-0 (PMC8460714; doi:10.1038/s41598-021-97600-0)
Supplement: Supplementary file 1 — Supplementary Information. [file 41598_2021_97600_MOESM1_ESM.docx]

Electronic supplementary material for

**The emerging vertebrate model species for neurophysiological studies is *Danionella cerebrum*, new species (Teleostei: Cyprinidae)**

Ralf Britz^1,2,*^, Kevin W. Conway^,3,4^, and Lukas Rüber^5,6^

^1^Senckenberg Natural History Collections Dresden, Museum of Zoology, DE-01109 Dresden, Germany. E-mail: [ralf.britz@senckenberg.de](mailto:ralf.britz@senckenberg.de).

^2^Research Associate, Department of Life Sciences, Natural History Museum, London, SW75BD, U.K.

^3^Department of Ecology and Conservation, Biology and Biodiversity Research and Teaching Collections, Texas A&M University, College Station, College Station, Texas 77543, USA

^4^Research Associate, Ichthyology, Australian Museum Research Institute, 1 William Street, Sydney, NSW 2010, Australia

^5^Naturhistorisches Museum Bern, 3005 Bern, Switzerland

^6^Aquatic Ecology and Evolution, Institute of Ecology and Evolution, University of Bern, 3012 Bern, Switzerland


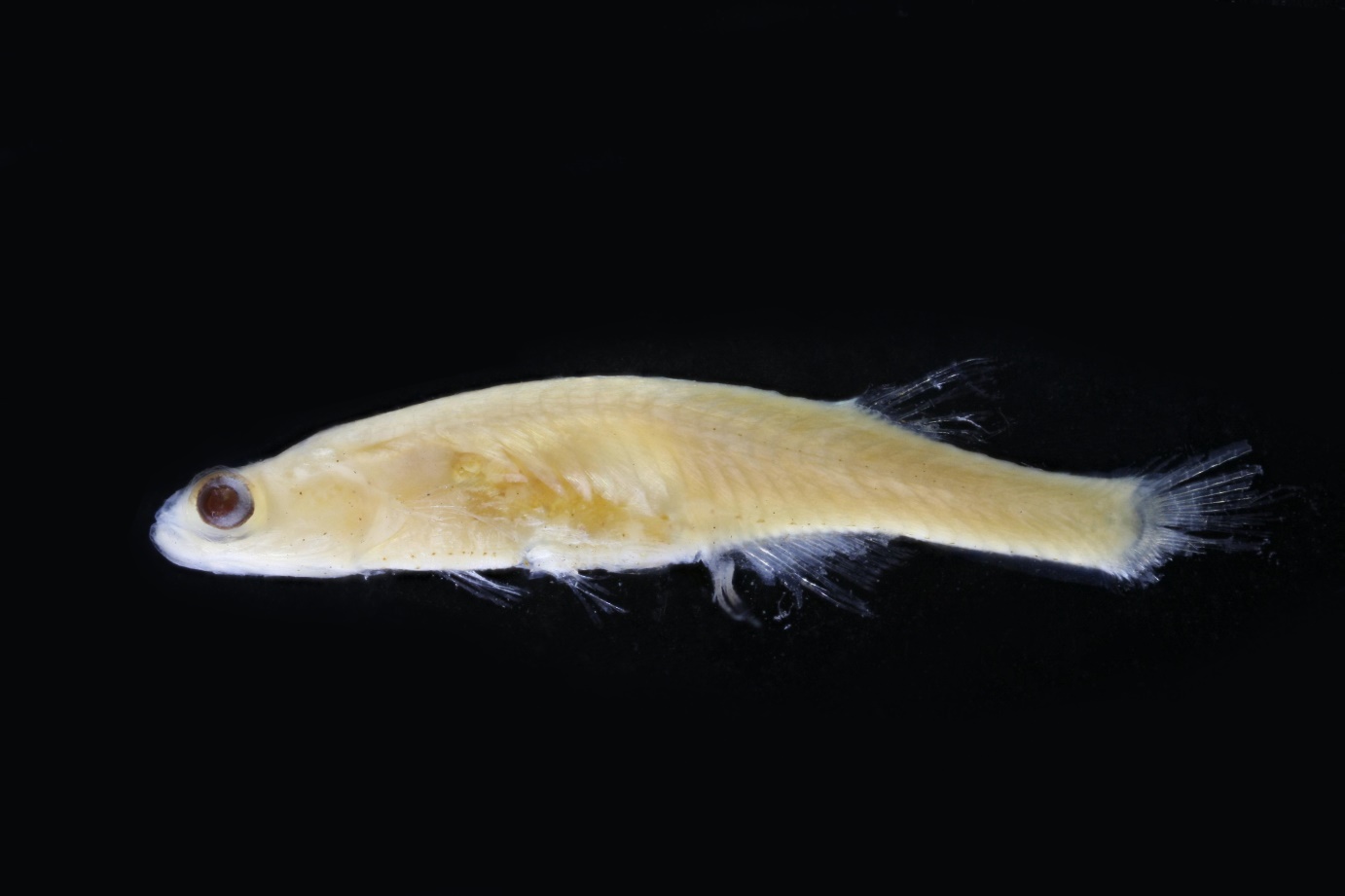


Supplementary Figure 1. Holotype of *Danionella translucida* (NRM 32232), 10.8 mm SL, in lateral view. Photo: Andrea Hennyey, NRM, Stockholm, Sweden.


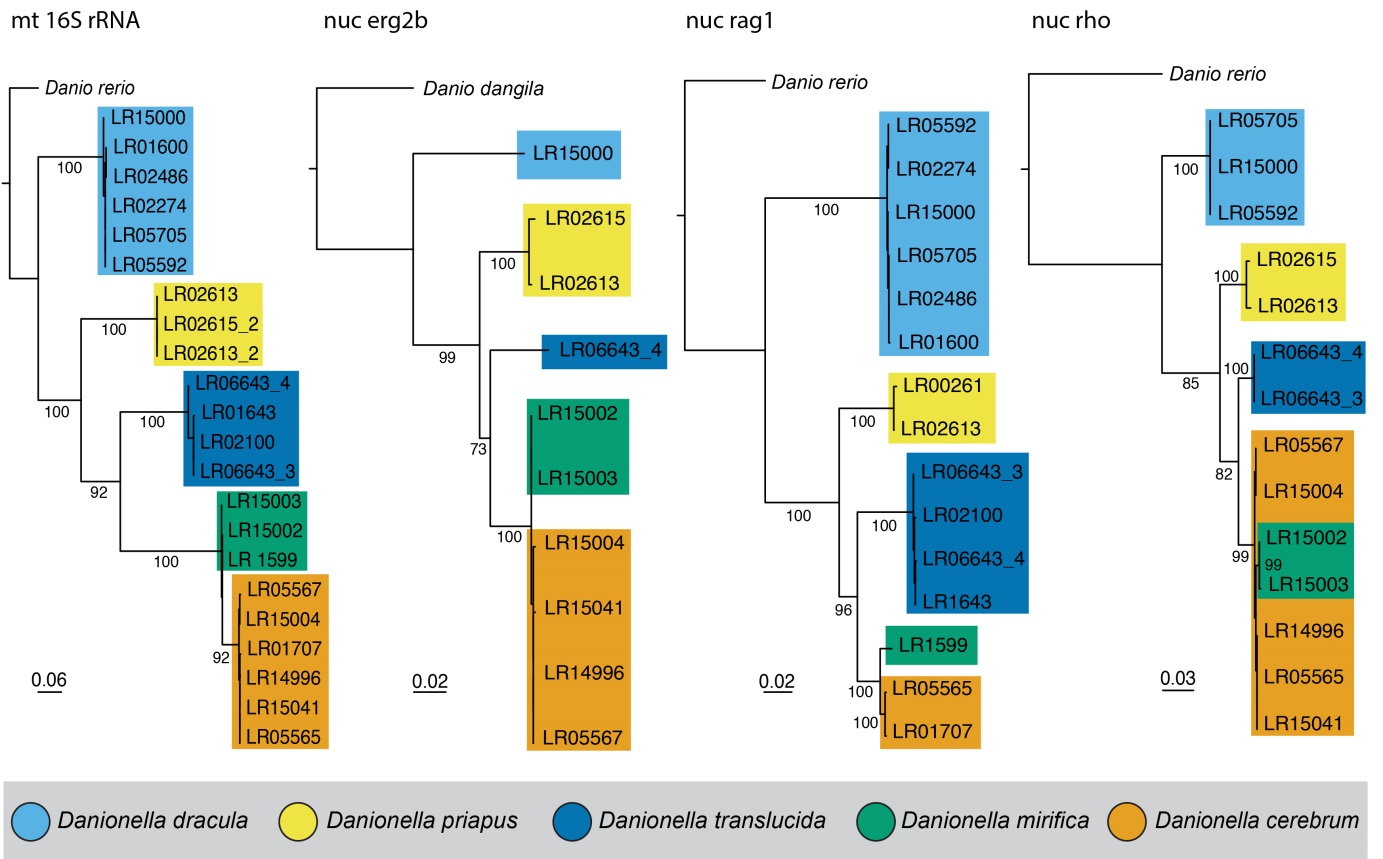


Supplementary Figure 2. Phylogenetic trees obtained from individual analyses of one mitochondrial and three nuclear genes.
